# Characterization of a novel HLA-A*11:335 allele resulting from a rare interlocus recombination involving HLA-A*11:01:01:01/126 and HLA-H*02:07/14/18 alleles with nanopore sequencing, in a volunteer from the China Marrow Donor Program

**DOI:** 10.1186/s12920-022-01176-1

**Published:** 2022-03-16

**Authors:** Li-Qun Zhang, Erik Rozemuller, Dan Wang, Xiang-Jun Liu, Jian-Ping Cai

**Affiliations:** 1 The Key Laboratory of Geriatrics, Beijing Institute of Geriatrics, Institute of Geriatric Medicine, Chinese Academy of Medical Sciences, Beijing Hospital/National Center of Gerontology of National Health Commission, No. 1 DaHua Road, Dong Dan, Beijing, People’s Republic of China; 2 GenDx, Genome Diagnostics B.V., Utrecht, the Netherlands; 3 Beijing BoFuRui Gene Diagnostic, LTD, Beijing, People’s Republic of China

**Keywords:** HLA-A*11:335, Interlocus recombination, NGS, Nanopore sequencing

## Abstract

**Background:**

The major histocompatibility complex (MHC) in humans includes three classical class I loci (A, B, and C), which are important biomarkers for the transplantation of organs and hematopoietic stem cells. In the MHC, polymorphism is known to be extremely high while interlocus recombination is rare. We report a rare interlocus recombination between HLA-A and HLA-H, which was analyzed using next generation sequencing and nanopore sequencing.

**Methods:**

In the sample, the genotypes of HLA-A, B, C, DRB1, and DQB1 were firstly determined using the methods of sequence-specific primer, sequence-specific oligonucleotide, Sanger’s sequencing, and NGS; however, HLA-A could not be phased. Nanopore sequencing was finally utilized to distinguish the sequence of the novel allele.

**Results:**

Finally, the novel HLA-A*11:335 allele was identified as an interlocus recombination involving HLA-A*11:01:01:01/126 and HLA-H*02:07/14/18 alleles; this was mainly achieved by nanopore sequencing.

**Conclusions:**

The identification of the interlocus recombination indicated that nanopore sequencing can be helpful in the characterization of novel alleles with complex rearrangements. Interlocus recombination has been identified as one of the mechanisms involved in the generation of novel HLA alleles.

**Supplementary Information:**

The online version contains supplementary material available at 10.1186/s12920-022-01176-1.

## Background

The main function of human classical class I loci (HLA-A, -B, and -C) is to display intracellularly digested foreign peptides (at antigen recognition site) to CD8 T cells [1]. For class I genes, exons 2, 3, and 4 encode the peptides of extracellular domains, α1, α2, and α3, respectively. The antigen recognition site is located in domains α1 and α2 [2, 3]. Exon 5 encodes the transmembrane domain of the protein. Mechanisms involved in the generation of human leucocyte antigen (HLA) polymorphism include crossing over, gene conversion, and point mutations [4]. Point mutations may produce synonymous or non-synonymous changes in protein level. The rate of non-synonymous changes is much higher than that of synonymous changes within antigen recognition site, indicating a selection-driven force [5]. In addition to point mutation, recombination between homologous genes has been involved in the generation of novel HLA alleles. Recombination involved in the same locus or different loci would result in intralocus or interlocus genomic recombinant, respectively [6]. We herein report a novel HLA-A*11 allele, A*11:335, which was identified as an interlocus recombination involving the HLA-A*11:01:01:01/126 and HLA-H*02:07/14/18 alleles in a Chinese bone marrow donor and analyzed the consequences of this recombination. This interlocus recombination was mainly characterized by nanopore sequencing.

## Methods

### Sample origination and first HLA typing

A total of 2964 specimens were sampled (approximately 2%) from the database of recruited volunteers of the China Marrow Donor Program in 2017 and subsequently genotyped for HLA-A, B, C, DRB1, and DQB1. The DNA sample 17ZZ2298 was originally extracted from a volunteer’s peripheral blood by the BGI laboratory, which was a cooperating partner of the China Marrow Donor Program. HLA typing for A, B, C, DRB1, and DQB1 was firstly performed with the BGI Next Generation Sequencing Typing method-RCHSBT (reliable, cost-effective and high-throughput sequence based typing) [7] (BGI, Shenzhen, China).

### HLA typing confirmation

HLA typing of the sample 17ZZ2298 was performed for a second time using the Sanger’s sequencing method (Shenzhen Tissue Bank Precision Medicine Co., Ltd., China) to examine the results acquired from RCHSBT. Because the result of HLA-A typing was A*01:01/11:126, which was different from that obtained by RCHSBT (A*01:01/11:01), the sample was typed for a third time using the sequence-specific oligonucleotide (SSO method) (Luminex 3D, Onelambda, California, USA). The result obtained with the SSO method was A*01:01/11:01, which was the same as that obtained using the RCHSBT method. The sample was further typed using Miseq based sequencing (Onelambda) and the assignment was A*01:01/11:126, with the following system comments: “Warning: mismatch in an intron, two or more variants cannot be phased, Locus has a high background position in exon.” The sample was further typed by next generation sequencing (NGS) using commercially available reagents (GenDX, Utrecht, The Netherlands) and a MiniSeq system (Illumina, San Diego, California, USA), and MinION based nanopore sequencing (ONT, Oxford, UK). Data were analyzed using the NGSengine software program (GenDX).

### Sequence blasting

The sequence of the novel allele (mismatched area) was blasted in the IMGT/HLA database using the “BlastN” tool.

### Transmembrane property analysis

The effects of the six missense mutations in exon 5 on the function of the transmembrane domain were analyzed and predicted with the PSIPRED online tool (http://bioinf.cs.ucl.ac.uk/psipred/). The amino acid sequences of exon 5 of HLA-A*11:335 and HLA-A*11:01:01:01 were entered into the online tool and analyzed.

## Results

### Genotype analysis

**Fig. 1:**
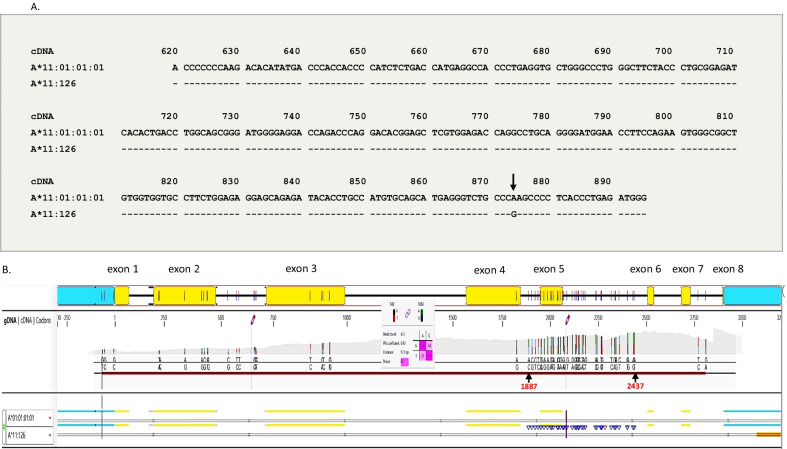
**A** Comparison of the exon 4 nucleotide sequence of HLA-A*11:126 and HLA-A*11:01:01:01. The numbers above the sequence indicate the cDNA position. Identity between sequences is indicated by dashes. The difference between sequences is highlighted by an arrowhead, which was the only difference between the two alleles. **B** A genotype analysis of HLA-A using the GENDX software program. The recommended genotype was A*01:01:01:01/A*11:126; however, numerous mismatches between exon 4 and exon 6 were observed (indicated by triangles). The purple vertical line insertion mark indicates an “AT” deletion in intron 5 in the A*11 allele. The yellow blocks indicate exon 1 ~ 8. The white blocks after each exon indicate the corresponding intron (1 ~ 7). The numbers below the yellow and white blocks indicate the position of gDNA (from 1 to 3000)

Sample 17ZZ2298 was firstly subjected to high-resolution typing of HLA-A, B, C, DRB1, and DQB1 using the BGI Next Generation Sequencing Typing method-RCHSBT [7]. Exon 1–7 of HLA-A, B and C, exon 1–3 of HLA-DRB1, and exon 2–3 of HLA-DQB1 were sequenced. The high-resolution HLA assignment of the sample was as follows: A*01:01:01, 11:01:01, B*15:32:01, 57:01:01, C*06:02:01, 12:03:01, DRB1*07:01:01, 12:01:01, and DQB1*03:01:01, 03:03:02; however, when the sample was further analyzed by the Sanger’s sequencing method using three different reagents (CSTB, Biocapital, and GenDx) the assignment for HLA-A was A*01:01/11:126. The sample was then reanalyzed by the SSO method and Miseq sequencing-based typing (Onelambda). The assignment determined by these methods was HLA-A*01:01/11:01 and HLA-A*01:01/11:126, respectively. However, the Miseq assignment had the following system comments: “Warning: mismatch in an intron, two or more variants cannot be phased.” Indicating the possibility that HLA-A*01:01/11:126 was not the correct assignment. The only difference between HLA-A*11:01:01:01 and HLA-A*11:126 was at c.874A > G in exon 4 (Fig. 1A).

The sample was then further analyzed by Miseq-based typing (GenDx). The data showed that there was a new allele, but exon 3 and exon 4 could not be phased with the MiSeq data. Therefore, the Miseq reads were further analyzed together with a low number of MiniON reads. The recommended genotype was HLA-A*01:01:01:01/A*11:126 (Fig. 1B); however, there were numerous mismatches between exon 4 and exon 6. All mismatches (indicated by blue or red triangles) were in the HLA-A*11 allele, and were located between the last heterozygous position in exon 4 (gDNA 1824) and intron 5 (gDNA 2437), which were heterozygous in this sample. The bases found at gDNA 1824 matched with the two reported HLA-A alleles. The first mismatched position in intron 4 (gDNA 1887) was heterozygous AC in this sample. All known HLA-A alleles have an A at this position. Thanks to the phasing information, we found that the C belonged to HLA-A*11new. The last two heterozygous positions (gDNA 2431 and gDNA 2437) had A-A in one allele and G-T in the other allele. A-A occurred in many HLA-A alleles while G-T was not present in any HLA-A alleles. When region 1887–2437 (matching with HLA-A*11:126) or region 1824–2437 (matching with HLA-A*11:01:01:01) were excluded, the data were an exact match with HLA-A. The typing results of HLA-A with each reagent and the final nomenclature are listed in Table 1.

**Table 1:** The typing results of sample 17ZZ2298 for HLA-A, B, C, DRB1, and DQB1 with different types of reagents and methods

Reagents	Methods	HLA-A		HLA-B		HLA-DRB1		HLA-C		HLA-DQB1	
BGI	Hiseq	01:01	11:01	15:32	57:01	07:01	12:01	06:02	12:03	03:01	03:03
CSTB	Sanger1	01:01	11:126	15:32	57:01	07:01	12:01	06:02	12:03	03:01	03:03
Biocapital	Sanger2	01:01	11:126								
Onelambda	SSO	01:01	11:01								
	Miseq1	01:01	11:126								
GenDx	Sanger3	01:01	11:126								
	Miseq2	01:01	**11:335**	**Final nomenclature**							
ONT	MinION										

The bold **11:335** and **final nomenclature** means: The name HLA-A*11:335 was officially assigned by the WHO Nomenclature Committee in May 2019

### Sequence blast and mutation analysis

**Fig. 2:**
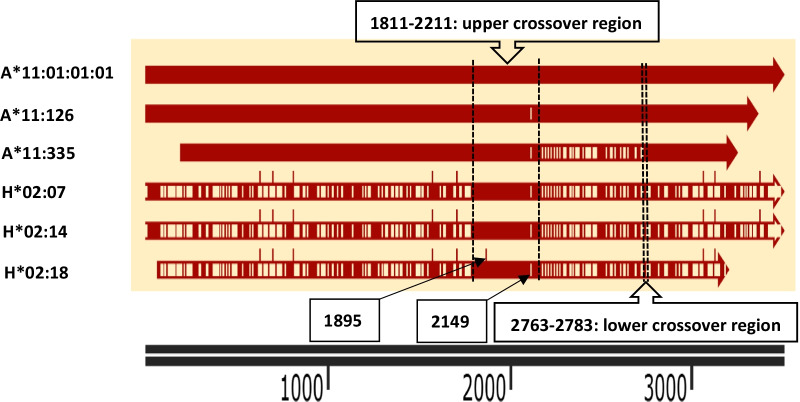
The alignment of the genomic sequence of HLA-A*11:01:01:01 with A*11:126, A*11:335, H*02:07, H*02:14, and H*02:18. The solid areas indicate those areas with an identical sequence to HLA-A*11:01:01:01. The blank areas indicate differences in the sequence. Regions 1811–2211 and 2763–2783 indicate the possible upper and lower crossover regions of HLA-A*11:01:01:01/126 and HLA-H*02:07/14/18

The sequence of region 1824–2437 (612 bp, because of an “AT” deletion in intron 5) was then blasted in the IMGT/HLA database. As shown in Table 2, 612/612 (100%) bases exactly matched with HLA-H*02:07/14/18(https://www.ebi.ac.uk/Tools/sss/ncbiblast/nucleotide.html). It was suggested that the sample contained a new HLA-A*11 allele (HLA-A*11:335), which was the result of the interlocus genomic exchange of HLA-A*11:01:01:01/126 and HLA-H*02:07/14/18. The distance between HLA-H and HLA-A on chromosome 6 is approximately 50 kb. The alignment of the genomic sequence of HLA-A*11:01:01:01 with A*11:126, A*11:335, H*02:07, H*02:14, and H*02:18 is shown in Fig. 2. The possible lower crossover region was located between 2763 and 2783, and the upper crossover region was located between 1811 and 2211. The detailed information is listed in the Additional file 1.

**Table 2:** The first five alleles of the blast results of the 612 bp fragment in the IMGT/HLA database

Align	DB:ID	Source	Length (bp)	Score (Bits)	Identities %
1	IMGTHLAgen: HLA27935	H*02:18	3144	1213.7	100
2	IMGTHLAgen: HLA22280	H*02:14	3502	1213.7	100
3	IMGTHLAgen: HLA22598	H*02:07	3502	1213.7	100
4	IMGTHLAgen: HLA23147	A*11:335	3071	1213.7	100
5	IMGTHLAgen: HLA27927	H*02:01:02	3142	1189.9	99.5

Blast results indicate that the 612 bp fragment could be found in HLA-H*02:18, HLA-H*02:14, HLA-H*02:07, and HLA-A*11:335 (submitted by our laboratory) with 100% identities in the IMGT/HLA database

### Transmembrane property analysis

**Fig. 3:**
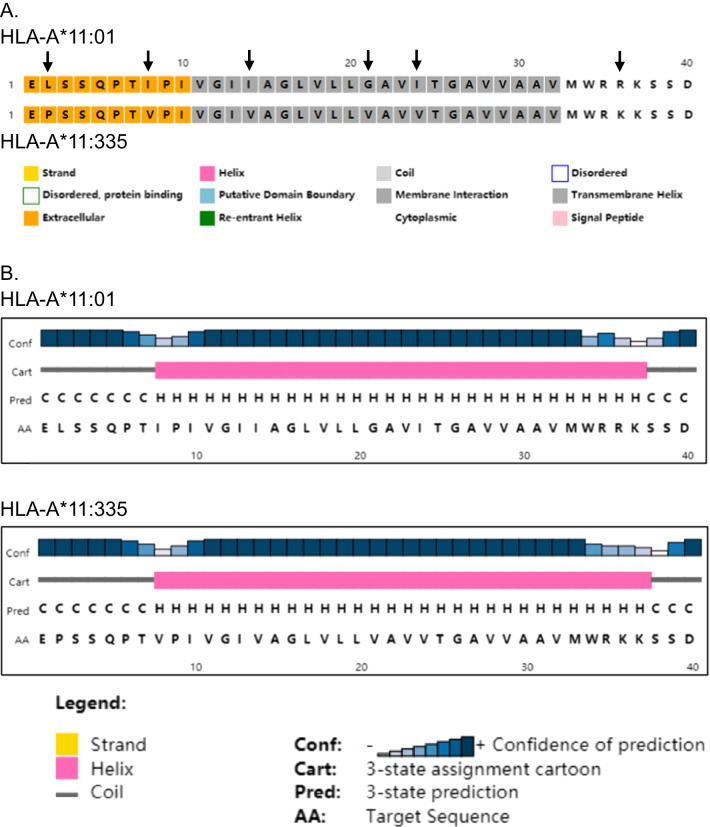
The effects of the six missense mutations on the property of the transmembrane domain of HLA-A*11:335. These six mutations did not lead to a destructive effect on the helix structure of the transmembrane domain. **A** Arrows indicate the difference of six amino acids (AA) in HLA-A*11:335 compared with HLA-A*11:01. Brown, gray, and white blocks indicate the extracellular, membrane interaction, and cytoplasmic domains, respectively. **B** The structure of the transmembrane domain in HLA-A*11:335 was little affected by the six AA substitutions compared with HLA-A*11:01. AA 8–38 remained as a helix (H) and AA 1–7 and 39–40 remained as coils (**C**) in HLA-A*11:335

As shown in Table 3, the sequence of HLA-A*11:335 differs from HLA-A*11:01:01:01 by 10 nucleotide substitutions, which resulted in three synonymous mutations and six missense mutations, mainly in exon 5. Exon 5 encodes the transmembrane domain of HLA-A. We analyzed the effects of the six missense mutations on the property of the transmembrane domain using the PSIPRED online tool (http://bioinf.cs.ucl.ac.uk/psipred/). The results showed that although six missense (three in the transmembrane domain) mutations were produced as a result of interlocus recombination between HLA-A and HLA-H, these mutations did not create destructive effects on the helix structure of the transmembrane domain (Fig. 3).

**Table 3:** Details of the base mutation and amino acid changes in exon 5 in the novel allele HLA-A*11:335 in comparison to HLA-A*11:01:01:01

Base position	899	900	916	934	951	956	964	987	990	1001
A*11:01:01:01	T	G	A	A	C	G	A	C	G	G
A*11:335	C	A	G	G	A	T	G	T	A	A
Codon change	CTG > CCA		ATC > GTC	ATT > GTT	CTC > CTA	GGA > GTA	ATC > GTC	GCC > GCT	GTG > GTA	AGG > AAG
AA. substitution	Leu > Pro		Ile > Val	Ile > Val	Leu = Leu	Gly > Val	Ile > Val	Ala = Ala	Val = Val	Arg > Lys
Codon position	276		282	288	293	295	298	305	306	310

### New allele nomenclature

The nucleotide sequence of the new allele has already been submitted to the DNA Data Bank of Japan (Accession No. LC474859) and to the IPD-IMGT/HLA Database [5, 8] (Submission No. HWS10054755). The name HLA-A*11:335 was officially assigned by the WHO Nomenclature Committee in May 2019. This follows the agreed policy that, subject to the conditions stated in the most recent *Nomenclature Report* [9], names will be assigned to new sequences as they are identified. The lists of these new names will be published in the next WHO Nomenclature Report.

## Discussion

A number of authors [10, 11] have proposed that interlocus recombination or gene conversion is an important mechanism in the maintenance of MHC polymorphism. A large portion of allelic variation in MHC loci is caused by variations in the antigen recognition site of exons 2 and 3. Furthermore, recombination or gene conversion cannot explain the high rate of nonsynonymous nucleotide substitution in comparison to the rate of synonymous nucleotide substitution. As previously suggested [12], the extremely high level of polymorphism at the MHC loci (80–90% heterozygosity) appears to be owing to over-dominant selection.

Based on our latest data, 191 alleles of the A locus were identified and A*11 was common (frequency: 23.203%) in Chinese volunteers [13]. In this sample, the HLA-A was analyzed with different reagents and methods (SSO, Sanger, NGS, and nanopore sequencing); however, the HLA-A sequence reads of this sample could not be matched to any previously described HLA-A allele (IPD-IMGT/HLA 3.35) without mismatches. A novel allele was suspected. Thanks to nanopore sequencing, the exact sequence of the novel allele (A*11:335) was determined.

The sequence of region 1824–2437 was then blasted in the IMGT/HLA database and the results showed that 612/612 bases matched exactly with HLA-H*02:07/14/18 (Table 2). It was suggested that the sample contained a new A*11 allele, which was the result of interlocus genomic exchange of HLA-A and HLA-H (see Additional file 1).

HLA-H is located between HLA-A and HLA-G, which are separated by less than 300 kb in the class I region of the MHC [14]. The paper of Paganini et al. [15] predicts protein structures based on HLA-H allele sequences and shows novel HLA-H alleles ranging from 18 amino-acids (AA) to 362 AA. Specific patterns of transmembrane HLA protein were found in two alleles: HLA-H*02:07 and HLA-H*02:14 (peptide signal, noncytoplasmic domain, transmembrane domain, cytoplasmic domain, glycosylation site, and a disulfide bond). The other 23 alleles lacked all or part of these critical domains and/or sites [8]. Gene conversion among loci is considered to be an important method for creating new HLA alleles [16]. Hughes [17] also indicates that interlocus recombination is a recurrent feature in the evolutionary history of the HLA class I region and suggests that class I pseudogenes arose through the duplication of class I genes over a long period of time. Because HLA-A and HLA-H are closely related, as well as in close proximity, it is possible that HLA-A enhances its diversity through gene conversion with HLA-H. Although Grimsley et al. suggests that the polymorphisms in HLA-H are not the result of interlocus gene conversion with HLA-A [18], our findings indicated that the polymorphisms in HLA-A may be partially due to interlocus gene conversion with HLA-H. The mechanism underlying the recombination between the two HLA loci is unknown.

The possible crossover regions and the possible involved HLA allele pairs were analyzed for this double crossover recombination. The lower crossover region was easier to determine, which should be located between 2763 and 2783. This was because there was a mutation (A) in A*11:01:01:01/126 before 2763 and a mutation (T) in H*02:07/14/18 after 2783, respectively, compared with A*11:335. The upper crossover region was not easily determined; several possibilities for this may exist. It was certain that the crossover point was after 1810. Four possible allele pairs and their upper crossover regions were: (a) HLA-A*11:01:01:01 versus HLA-H*02:07/14 in 1811–2148; (b) HLA-A*11:126 versus HLA-H*02:07/14 in 1811–2211; (c) HLA-A*11:01:01:01 versus HLA-H*02:18 in 1896–2148; (d) HLA-A*11:126 versus HLA-H*02:18 in 1896–2211. The exact panel of the interlocus recombination may be resolved by sequencing of the HLA region of the donor’s parents. Detailed information is listed in the Additional file 1.

The sequence of A*11:335 differed from HLA-A*11:01:01:01 by 10 nucleotide substitutions, resulting in three synonymous mutations and six missense mutations in exon 5 (Table 3). Exon 5 encoded the transmembrane domain of HLA-A. The effects of the six missense mutations on the property of the transmembrane domain were analyzed using the PSIPRED online tool. The result showed that although six missense (three in the transmembrane domain) mutations were produced due to interlocus recombination between HLA-A and HLA-H, these mutations did not lead to a destructive effect on the helix structure of the transmembrane domain (Fig. 3). The mechanism and the consequence of this interlocus recombination remain largely unknown.

## Conclusions

A novel HLA-A*11:335 allele, as an interlocus recombination involving the HLA-A*11:01:01:01/126 and HLA-H*02:07/14/18 alleles, was identified in a volunteer from the China Marrow Donor Program. The results indicated that nanopore sequencing can be helpful in the characterization of novel alleles with complex rearrangements.

## Supplementary Information

**Additional file 1.** The detailed alignment of the genomic sequence of HLA-A*11:01:01:01 with A*11:126, A*11:335, H*02:07, H*02:14, and H*02:18. The parts highlighted in yellow are identical to the sequence in HLA-A*11:335 and HLA-H*02:07/14/18. The first line "Untitled" sequence (virtual) was the common sequence of the aligned alleles, which could cover the total length of all the sequences. “Red nucleotide” means the nucleotide was a mutation at this position in the aligned sequences.

## Data Availability

The sequences of the novel allele described in this manuscript have been previously uploaded to the public dbVar, DNA Data Bank of Japan (Accession No. LC474859.1), and to the IPD-IMGT/HLA Database [8] for nomenclature. The details of the submission and the whole sequence of HLA-A*11:335 can be found at https://www.ncbi.nlm.nih.gov/dbvar/studies/nstd217/, http://getentry.ddbj.nig.ac.jp/getentry/na/LC474859 or https://www.ncbi.nlm.nih.gov/nuccore/LC474859.

## Abbreviations

HLA: Human leucocyte antigen
NGS: Next generation sequencing
MHC: Major histocompatibility complex
SSP: Sequence-specific primer
SSO: Sequence-specific oligonucleotide
DNA: Deoxy-ribo nucleic acid
ONT: Oxford nanopore technology
RCHSBT: Reliable, cost-effective and high-throughput sequence based typing
MiniON: Mini oxford nanopore
IMGT: International ImMunoGeneTics
SNPs: Single nucleotide polymorphisms
kb: Kilobases
PSIPRED: Predict Secondary Structure
IPD: Immuno Polymorphism Database
WHO: World Health Organization
