# Supplementary material for: Characterization of a novel HLA-A*11:335 allele resulting from a rare interlocus recombination involving HLA-A*11:01:01:01/126 and HLA-H*02:07/14/18 alleles with nanopore sequencing, in a volunteer from the China Marrow Donor Program
Source: BMC Med Genomics. 2022 Mar 16;15:58. doi: 10.1186/s12920-022-01176-1 (PMC8925214; doi:10.1186/s12920-022-01176-1)
Supplement: Supplementary file 1 — Additional file 1. The detailed alignment of the genomic sequence of HLA-A*11:01:01:01 with A*11:126, A*11:335, H*02:07, H*02:14, and H*02:18. The parts highlighted in yellow are identical to the sequence in HLA-A*11:335 and HLA-H*02:07/14/18. The first line "Untitled" sequence (virtual) was the common sequence of the aligned alleles, which could cover the total length of all the sequences. “Red nucleotide” means the nucleotide was a mutation at this position in the aligned sequences. [file 12920_2022_1176_MOESM1_ESM.pdf]

Page 1 HLA-A1101-126-135-H02-07-14-18

"Untitled" was the common sequence (virtual) of the aligned alleles, which could cover the total length of all the sequences. "Red nucleotide" means the nucleotide at this position was a mutation in the aligned sequences.

```
             1
Untitled ... GCACAAGAGCAGAGGGGTCAGGGCGAAGTCCCAGGGCCCCAGGCGTGGCTCTCAGGGTCTCAGGCCCCGAAGGCGGTGTATG
A11-01010... ---CAAGAGCAGAGGGGTCAGGGCGAAGTCCCAGGGCCCCAGGCGTGGCTCTCAGGGTCTCAGGCCCCGAAGGCGGTGTATG
A11-126 (... ---CAAGAGCAGAGGGGTCAGGGCGAAGTCCCAGGGCCCCAGGCGTGGCTCTCAGGGTCTCAGGCCCCGAAGGCGGTGTATG
A11-335 (... ----------------------------------------------------------------------------------
H02-07 (H... GCACAGGAGGAGCGGGGTCAGGGCGAAGTCCCAGGGCCCCAGGCGTGGCTCTCAGGGTCTCAGGCCCCGAAGGCGGTGTATG
H02-14 (H... GCACAGGAGGAGCGGGGTCAGGGCGAAGTCCCAGGGCCCCAGGCGTGGCTCTCAGGGTCTCAGGCCCCGAAGGCGGTGTATG
H02-18 (H... -------------------------------------------------------------------CGAAGGCGGTGTATG

...............................................................................................

             83                                                                             164
Untitled ... GATTGGGGATGCCCCGCCTTGGGGATTCGCCACCTCCGCAGTTTCTCTTCTCCCTCTCACAACCTGCGACGGGTCCTTCTTC
A11-01010... GATTGGGGAGTCCCAGCCTTGGGGATTCCCCCAACTCCGCAGTTTCTTTTCTCCCTCTCCCAACCTACGTAGGGTCCTTCATC
A11-126 (... GATTGGGGAGTCCCAGCCTTGGGGATTCCCCCAACTCCGCAGTTTCTTTTCTCCCTCTCCCAACCTACGTAGGGTCCTTCATC
A11-335 (... ----------------------------------------------------------------------------------
H02-07 (H... GATTGGGGATGCCCCGCCTTGGGGATTCGCCACCTCCGCAGTTTCTCTTCT---TCTCACAACCTGCGACGGGTCCTTCTTC
H02-14 (H... GATTGGGGATGCCCCGCCTTGGGGATTCGCCACCTCCGCAGTTTCTCTTCT---TCTCACAACCTGCGACGGGTCCTTCTTC
H02-18 (H... GATTGGGGATGCCCCGCCTTGGGGATTCGCCACCTCCGCAGTTTCTCTTCT---TCTCACAACCTGCGACGGGTCCTTCTTC

...............................................................................................

             165                                                                            246
Untitled ... CTCGATACTCACGAAGCGGACACAGTTCTCATTCCCACTAGGTGTCGGGTTTCCAGAGAAGCCAATCAGTGCCTCCGCGGTC
A11-01010... CTGGATACTCACGACGCGGACCCAGTTCTCACTCCCATTGGGTGTCGGGTTTCCAGAGAAGCCAATCAGTGTCTTCGCGGTC
A11-126 (... CTGGATACTCACGACGCGGACCCAGTTCTCACTCCCATTGGGTGTCGGGTTTCCAGAGAAGCCAATCAGTGTCTTCGCGGTC
A11-335 (... --------------------------------TCCCATTGGGTGTCGGGTTTCCAGAGAAGCCAATCAGTGTCTTCGCGGTC
H02-07 (H... CTCGATACTCACGAAGCGGACACAGTTCTCATTCCCACTAGGTGTCGGGTTTCTAGAGAAGCCAATCGGTGCCGCCGCGGTC
H02-14 (H... CTCGATACTCACGAAGCGGACACAGTTCTCATTCCCACTAGGTGTCGGGTTTCTAGAGAAGCCAATCGGTGCCGCCGCGGTC
H02-18 (H... CTCGATACTCACGAAGCGGACACAGTTCTCATTCCCACTAGGTGTCGGGTTTCTAGAGAAGCCAATCGGTGCCGCCGCGGTC

...............................................................................................

             247                                                                            328
Untitled ... CCTCTTCTAAAGTCCCCACGCACCCACCGGGACTCAGATTCTCCCCAGACCCCGAGGATGGCCCTCATGGCGCCCCGAACCC
A11-01010... GCTCTTCTAAAGTCCGCACGCACCCACCGGGACTCAGATTCTCCCCAGACCCCGAGGATGGCCGTCATGGCGCCCCGAACCC
A11-126 (... GCTCTTCTAAAGTCCGCACGCACCCACCGGGACTCAGATTCTCCCCAGACCCCGAGGATGGCCGTCATGGCGCCCCGAACCC
A11-335 (... GCTCTTCTAAAGTCCGCACGCACCCACCGGGACTCAGATTCTCCCCAGACCCCGAGGATGGCCGTCATGGCGCCCCGAACCC
H02-07 (H... CCGGTTCTAAAGTCCCCACGCACCCACCGGGACTCAGATTCTCCCCAGACGCCGAGGATGGTGCTCATGGCGCCCCGAACCC
H02-14 (H... CCGGTTCTAAAGTCCCCACGCACCCACCGGGACTCAGATTCTCCCCAGACGCCGAGGATGGTGCTCATGGCGCCCCGAACCC
H02-18 (H... CCGGTTCTAAAGTCCCCACGCACCCACCGGGACTCAGATTCTCCCCAGACGCCGAGGATGGTGCTCATGGCGCCCCGAACCC

...............................................................................................

             329                                                                            410
Untitled ... TCCTCCTGCTACTCTCAGGGGCCCTGGCCCTGACCCAGACCTGGGCGCGTGAGTGCAGGGTCTGCAGGGAAACCGCCTCTGC
A11-01010... TCCTCCTGCTACTCTCGGGGGCCCTGGCCCTGACCCAGACCTGGGCGGGTGAGTGCGGGGTCGGGAGGGAAACCGCCTCTGC
A11-126 (... TCCTCCTGCTACTCTCGGGGGCCCTGGCCCTGACCCAGACCTGGGCGGGTGAGTGCGGGGTCGGGAGGGAAACCGCCTCTGC
A11-335 (... TCCTCCTGCTACTCTCGGGGGCCCTGGCCCTGACCCAGACCTGGGCGGGTGAGTGCGGGGTCGGGAGGGAAACCGCCTCTGC
H02-07 (H... TCCTCCTGCTGCTCTCAGGGGCCCTGGCCCTGACCCAGACCTGGGCGCGTGAGTGCAGGGTCTGCAGGGAAATGGTC-----
H02-14 (H... TCCTCCTGCTGCTCTCAGGGGCCCTGGCCCTGACCCAGACCTGGGCGCGTGAGTGCAGGGTCTGCAGGGAAATGGTC-----
H02-18 (H... TCCTCCTGCTGCTCTCAGGGGCCCTGGCCCTGACCCAGACCTGGGCGCGTGAGTGCAGGGTCTGCAGGGAAATGGTC-----

...............................................................................................

             411                                                                            492
Untitled ... GGGGAGAAGCAAGGGGCCCTCCCGGCGGGGGCGCAGGACCCAGGGAGCCGCGCAGGGAGGAGGGTCGGGCAGGTCTCAGCCA
A11-01010... GGGGAGAAGCAAGGGGCCCTCCTGGCGGGGGCGCAGGACCGGGGGAGCCGCGCCGGGAGGAGGGTCGGGCAGGTCTCAGCCA
A11-126 (... GGGGAGAAGCAAGGGGCCCTCCTGGCGGGGGCGCAGGACCGGGGGAGCCGCGCCGGGAGGAGGGTCGGGCAGGTCTCAGCCA
A11-335 (... GGGGAGAAGCAAGGGGCCCTCCTGGCGGGGGCGCAGGACCGGGGGAGCCGCGCCGGGAGGAGGGTCGGGCAGGTCTCAGCCA
H02-07 (H... -GGGAGGAGCGAGGGGCCCGCCCGGCGGGGGCGCAGGACCCAGGGAGCCGCGCAGGGAGGAGGGTCGGGCGGGTCTCAGCTC
H02-14 (H... -GGGAGGAGCGAGGGGCCCGCCCGGCGGGGGCGCAGGACCCAGGGAGCCGCGCAGGGAGGAGGGTCGGGCGGGTCTCAGCTC
H02-18 (H... -GGGAGGAGCGAGGGGCCCGCCCGGCGGGGGCGCAGGACCCAGGGAGCCGCGCAGGGAGGAGGGTCGGGCGGGTCTCAGCTC

...............................................................................................
```

```
               493                                                                              574
Untitled ...   CTCCTCGCCCCCAGGCTCCCACTCCATGAGGTATTTCTACACCACCATGTCCCGGCCCGGCCGCGGGGAGCCCCGCTTCATC
A11-01010...   CTGCTCGCCCCCAGGCTCCCACTCCATGAGGTATTTCTACACCTCCGTGTCCCGGCCCGGCCGCGGGGAGCCCCGCTTCATC
A11-126 (...   CTGCTCGCCCCCAGGCTCCCACTCCATGAGGTATTTCTACACCTCCGTGTCCCGGCCCGGCCGCGGGGAGCCCCGCTTCATC
A11-335 (...   CTGCTCGCCCCCAGGCTCCCACTCCATGAGGTATTTCTACACCTCCGTGTCCCGGCCCGGCCGCGGGGAGCCCCGCTTCATC
H02-07 (H...   CTCCTCGCTCCCAGGTTCCCACTCCATGAGGTATTTCTACACCACCATGTCCCGGCCCGGCCGCGGGGAGCCCCGCTTCATC
H02-14 (H...   CTCCTCGCTCCCAGGTTCCCACTCCATGAGGTATTTCTACACCACCATGTCCCGGCCCGGCCGCGGGGAGCCCCGCTTCATC
H02-18 (H...   CTCCTCGCTCCCAGGTTCCCACTCCATGAGGTATTTCTACACCACCATGTCCCGGCCCGGCCGCGGGGAGCCCCGCTTCATC

...................................................................................................

               575                                                                              656
Untitled ...   TCCGTCGGCTACGTGGACGACACGCAGTTCGTGCGGTTCGACAGCGACGACGCGAGCCAGAGAAAGGAGCCGCGGGCGCCGT
A11-01010...   GCCGTGGGCTACGTGGACGACACGCAGTTCGTGCGGTTCGACAGCGACGCCGCGAGCCAGAGGATGGAGCCGCGGGCGCCGT
A11-126 (...   GCCGTGGGCTACGTGGACGACACGCAGTTCGTGCGGTTCGACAGCGACGCCGCGAGCCAGAGGATGGAGCCGCGGGCGCCGT
A11-335 (...   GCCGTGGGCTACGTGGACGACACGCAGTTCGTGCGGTTCGACAGCGACGCCGCGAGCCAGAGGATGGAGCCGCGGGCGCCGT
H02-07 (H...   TCCGTCGGCTACGTGGACGATACGCAGTTCGTGCGGTTCGACAGCGACGACGCGAGTCCGAGAGAGGAGCCGCGGGCGCCGT
H02-14 (H...   TCCGTCGGCTACGTGGACGATACGCAGTTCGTGCGGTTCGACAGCGACGACGCGAGTCCGAGAGAGGAGCCGCGGGCGCCGT
H02-18 (H...   TCCGTCGGCTACGTGGACGATACGCAGTTCGTGCGGTTCGACAGCGACGACGCGAGTCCGAGAGAGGAGCCGCGGGCGCCGT

...................................................................................................

               657                                                                              738
Untitled ...   GGATAGAGCAGGAGGGGCCAAAGTATTGGGACCAGAACACACAGAACTTCAAGGCCCAGTCACAGACTGAACGAGAGAACCT
A11-01010...   GGATAGAGCAGGAGGGGCCGGAGTATTGGGACCAGGAGACACGGAATGTGAAGGCCCAGTCACAGACTGACCGAGTGGACCT
A11-126 (...   GGATAGAGCAGGAGGGGCCGGAGTATTGGGACCAGGAGACACGGAATGTGAAGGCCCAGTCACAGACTGACCGAGTGGACCT
A11-335 (...   GGATAGAGCAGGAGGGGCCGGAGTATTGGGACCAGGAGACACGGAATGTGAAGGCCCAGTCACAGACTGACCGAGTGGACCT
H02-07 (H...   GGATGGAGCGGGAGGGGCCAAAGTATTGGGACCGGAACACACAGATCTGCAAGGCCCAGGCACAGACTGAACGAGAGAACCT
H02-14 (H...   GGATGGAGCGGGAGGGGCCAAAGTATTGGGACCGGAACACACAGATCTGCAAGGCCCAGGCACAGACTGAACGAGAGAACCT
H02-18 (H...   GGATGGAGCGGGAGGGGCCAAAGTATTGGGACCGGAACACACAGATCTGCAAGGCCCAGGCACAGACTGAACGAGAGAACCT

...................................................................................................

               739                                                                              820
Untitled ...   GCGGACCCCGCTCCGCTACTACAACCAGAGCGAGGACGGTGAGTGACCCCGGCCCGGGGCGCAGGTCACGACCCCTCCCCAT
A11-01010...   GGGGACCCTGCGCGGCTACTACAACCAGAGCGAGGACGGTGAGTGACCCCGGCCCGGGGCGCAGGTCACGACCCCT---CAT
A11-126 (...   GGGGACCCTGCGCGGCTACTACAACCAGAGCGAGGACGGTGAGTGACCCCGGCCCGGGGCGCAGGTCACGACCCCT---CAT
A11-335 (...   GGGGACCCTGCGCGGCTACTACAACCAGAGCGAGGACGGTGAGTGACCCCGGCCCGGGGCGCAGGTCACGACCCCT---CAT
H02-07 (H...   GCGGATCGCGCTCCGCTACTACAACCAGAGCGAGGGCGGTGAGTGACCCCGGCCCGGGGCGCAGGTCACGACCCCTCCCCAT
H02-14 (H...   GCGGATCGCGCTCCGCTACTACAACCAGAGCGAGGGCGGTGAGTGACCCCGGCCCGGGGCGCAGGTCACGACCCCTCCCCAT
H02-18 (H...   GCGGATCGCGCTCCGCTACTACAACCAGAGCGAGGGCGGTGAGTGACCCCGGCCCGGGGCGCAGGTCACGACCCCTCCCCAT

...................................................................................................

               821                                                                              902
Untitled ...   CCCCCACGGACGGGCCAGGTCGCCCACAGTCTCCGGGTCCGAGATCCACCCCGAAACCGCGGGACCCCGAGACCCTTGACCC
A11-01010...   CCCCCACGGACGGGCCAGGTGGCCCACAGTCTCCGGGTCCGAGATCCACCCCGAAGCCGCGGGACCCCGAGACCCTTGCCCC
A11-126 (...   CCCCCACGGACGGGCCAGGTGGCCCACAGTCTCCGGGTCCGAGATCCACCCCGAAGCCGCGGGACCCCGAGACCCTTGCCCC
A11-335 (...   CCCCCACGGACGGGCCAGGTGGCCCACAGTCTCCGGGTCCGAGATCCACCCCGAAGCCGCGGGACCCCGAGACCCTTGCCCC
H02-07 (H...   CCCCCACGGA-GGGCCGGGTCGCCTCGAGTCTCTGGGTCCGAGATCCACCCCGAAACCGCGGGATCCCGAGACCCTTGACCT
H02-14 (H...   CCCCCACGGA-GGGCCGGGTCGCCTCGAGTCTCTGGGTCCGAGATCCACCCCGAAACCGCGGGATCCCGAGACCCTTGACCT
H02-18 (H...   CCCCCACGGA-GGGCCGGGTCGCCTCGAGTCTCTGGGTCCGAGATCCACCCCGAAACCGCGGGATCCCGAGACCCTTGACCT

...................................................................................................

               903                                                                              984
Untitled ...   GGGAGAGGCCCAGGCGCCTTTACCCGGTTTCATTTTCAGTTTAGGCCAAAAATCCCCCCGGGTTGGTCGGGGCCGGGCAGGG
A11-01010...   GGGAGAGGCCCAGGCGCCTTTACCCGGTTTCATTTTCAGTTTAGGCCAAAAATCCCCCCGGGTTGGTCGGGGCCGGGCAGGG
A11-126 (...   GGGAGAGGCCCAGGCGCCTTTACCCGGTTTCATTTTCAGTTTAGGCCAAAAATCCCCCCGGGTTGGTCGGGGCCGGGCAGGG
A11-335 (...   GGGAGAGGCCCAGGCGCCTTTACCCGGTTTCATTTTCAGTTTAGGCCAAAAATCCCCCCGGGTTGGTCGGGGCCGGGCAGGG
H02-07 (H...   GGGAGAGGCCCAGGCGCCTTTACCCGGTTTCATTTTCAGTTTAGGCC-AAAATCCCCGCGGGTTGGTCGGGGCGGGGCTGGG
H02-14 (H...   GGGAGAGGCCCAGGCGCCTTTACCCGGTTTCATTTTCAGTTTAGGCC-AAAATCCCCGCGGGTTGGTCGGGGCGGGGCTGGG
H02-18 (H...   GGGAGAGGCCCAGGCGCCTTTACCCGGTTTCATTTTCAGTTTAGGCC-AAAATCCCCGCGGGTTGGTCGGGGCGGGGCTGGG

...................................................................................................
```

```
                985                                                                             1066
Untitled ...    CTCGGGGGACCGGGCTGACCGCGGGGTCGGGGCCAGGTTCTCACACCATCCAGATAATGTATGGCTGCGACGTGGGGCCCGA
A11-01010...    CTTGGGGGACTGGGCTGACCGCGGGGTCGGGGCCAGGTTCTCACACCATCCAGATAATGTATGGCTGCGACGTGGGGCCGGA
A11-126 (...    CTTGGGGGACTGGGCTGACCGCGGGGTCGGGGCCAGGTTCTCACACCATCCAGATAATGTATGGCTGCGACGTGGGGCCGGA
A11-335 (...    CTTGGGGGACTGGGCTGACCGCGGGGTCGGGGCCAGGTTCTCACACCATCCAGATAATGTATGGCTGCGACGTGGGGCCGGA
H02-07 (H...    CTCGGGGGACCGGGCTGACCGCGGGGGCGGGGCCAGGTTCTCACACCATGCAGGTGATGTATGGCTGCGACGTGGGGCCCGA
H02-14 (H...    CTCGGGGGACCGGGCTGACCGCGGGGGCGGGGCCAGGTTCTCACACCATGCAGGTGATGTATGGCTGCGACGTGGGGCCCGA
H02-18 (H...    CTCGGGGGACCGGGCTGACCGCGGGGGCGGGGCCAGGTTCTCACACCATGCAGGTGATGTATGGCTGCGACGTGGGGCCCGA

..................................................................................................

                1067                                                                            1148
Untitled ...    CGGGCGCTTCCTCCGCGGGTACCAACAGCACGCCTACGACGGCAAGGATTACATCGCCCTGAACGAGGACCTGCGCTCCTGG
A11-01010...    CGGGCGCTTCCTCCGCGGGTACCGGCAGGACGCCTACGACGGCAAGGATTACATCGCCCTGAACGAGGACCTGCGCTCTTGG
A11-126 (...    CGGGCGCTTCCTCCGCGGGTACCGGCAGGACGCCTACGACGGCAAGGATTACATCGCCCTGAACGAGGACCTGCGCTCTTGG
A11-335 (...    CGGGCGCTTCCTCCGCGGGTACCGGCAGGACGCCTACGACGGCAAGGATTACATCGCCCTGAACGAGGACCTGCGCTCTTGG
H02-07 (H...    CGGGCGCTTCCTCCGCGGGTATGAACAGCACGCCTACGACGGCAAGGATTACATCGCCCTGAACGAGGACCTGCGCTCCTGG
H02-14 (H...    CGGGCGCTTCCTCCGCGGGTATGAACAGCACGCCTACGACGGCAAGGATTACATCGCCCTGAACGAGGACCTGCGCTCCTGG
H02-18 (H...    CGGGCGCTTCCTCCGCGGGTATGAACAGCACGCCTACGACGGCAAGGATTACATCGCCCTGAACGAGGACCTGCGCTCCTGG

..................................................................................................

                1149                                                                            1230
Untitled ...    ACCGCGGCGGACATGGCAGCTCAGATCACCAAGCGCAAGTGGGAGGCGGCCCATCCGGCGGAGCAGCAGAGAGCCTACCTGG
A11-01010...    ACCGCGGCGGACATGGCAGCTCAGATCACCAAGCGCAAGTGGGAGGCGGCCCATGCGGCGGAGCAGCAGAGAGCCTACCTGG
A11-126 (...    ACCGCGGCGGACATGGCAGCTCAGATCACCAAGCGCAAGTGGGAGGCGGCCCATGCGGCGGAGCAGCAGAGAGCCTACCTGG
A11-335 (...    ACCGCGGCGGACATGGCAGCTCAGATCACCAAGCGCAAGTGGGAGGCGGCCCATGCGGCGGAGCAGCAGAGAGCCTACCTGG
H02-07 (H...    ACCGCGGCGGACATGGCAGCTCAGATCACCAAGCGCAAGTGGGAGGCGGCCCGTCGGGCGGAGCAGCGGAGAGTCTACCTGG
H02-14 (H...    ACCGCGGCGGACATGGCAGCTCAGATCACCAAGCGCAAGTGGGAGGCGGCCCGTCGGGCGGAGCAGCGGAGAGTCTACCTGG
H02-18 (H...    ACCGCGGCGGACATGGCAGCTCAGATCACCAAGCGCAAGTGGGAGGCGGCCCGTCGGGCGGAGCAGCTGAGAGCCTACCTGG

..................................................................................................

                1231                                                                            1312
Untitled ...    AGGGCCAGTTCGTGGAGTGGCTCCGCAGATACCTGGAGAACGGGAAGGAGACGCTGCAGCGCACGGGTACCAGGGGCCACAG
A11-01010...    AGGGCCGGTGCGTGGAGTGGCTCCGCAGATACCTGGAGAACGGGAAGGAGACGCTGCAGCGCACGGGTACCAGGGGCCACGG
A11-126 (...    AGGGCCGGTGCGTGGAGTGGCTCCGCAGATACCTGGAGAACGGGAAGGAGACGCTGCAGCGCACGGGTACCAGGGGCCACGG
A11-335 (...    AGGGCCGGTGCGTGGAGTGGCTCCGCAGATACCTGGAGAACGGGAAGGAGACGCTGCAGCGCACGGGTACCAGGGGCCACGG
H02-07 (H...    AGGGCGAGTTCGTGGAGTGGCTCCGCAGATACCTGGAGAACGGGAAGGAGACGCTGCAGCGCGCGGGTACCAGGGGCCACAG
H02-14 (H...    AGGGCGAGTTCGTGGAGTGGCTCCGCAGATACCTGGAGAACGGGAAGGAGACGCTGCAGCGCACGGGTACCAGGGGCCACAG
H02-18 (H...    AGGGCGAGTTCGTGGAGTGGCTCCGCAGATACCTGGAGAACGGGAAGGAGACGCTGCAGCGCGCGGGTACCAGGGGCCACAG

..................................................................................................

                1313                                                                            1394
Untitled ...    GGCGCCTCCCTGATCGCCTATAGATCTCCCGGGCTGGCCTCCCACAAGAAAGGAGACAAATGGGACCAACACTATAATATC
A11-01010...    GGCGCCTCCCTGATCGCCTATAGATCTCCCGGGCTGGCCTCCCACAAGGAGGGGAGACAATTGGGACCAACACTAGAATATC
A11-126 (...    GGCGCCTCCCTGATCGCCTATAGATCTCCCGGGCTGGCCTCCCACAAGGAGGGGAGACAATTGGGACCAACACTAGAATATC
A11-335 (...    GGCGCCTCCCTGATCGCCTATAGATCTCCCGGGCTGGCCTCCCACAAGGAGGGGAGACAATTGGGACCAACACTAGAATATC
H02-07 (H...    GGCGCCTCCCTGATCGCCTGTAGATCTCCGGGGCTGGCCTCCCACAAGAAAGGAGACAAATGGGACCAACACTATAATATC
H02-14 (H...    GGCGCCTCCCTGATCGCCTGTAGATCTCCGGGGCTGGCCTCCCACAAGAAAGGAGACAAATGGGACCAACACTATAATATC
H02-18 (H...    GGCGCCTCCCGGATCGCCTGTAGATCTCCGGGGCTGGCCTCCCACAAGAAAGGAGACAAATGGGACCAACACTATAATATC

..................................................................................................

                1395                                                                            1476
Untitled ...    ACCCTCCCTCTGGTCCTGAGGGAGAAGAATCCTCCTGGGTTTCCAGATCCTGTACCAGAGAGTGACTCTGAGGTTCCGCCCT
A11-01010...    ACCCTCCCTCTGGTCCTGAGGGAGAGGAATCCTCCTGGGTTTCCAGATCCTGTACCAGAGAGTGACTCTGAGGTTCCGCCCT
A11-126 (...    ACCCTCCCTCTGGTCCTGAGGGAGAGGAATCCTCCTGGGTTTCCAGATCCTGTACCAGAGAGTGACTCTGAGGTTCCGCCCT
A11-335 (...    ACCCTCCCTCTGGTCCTGAGGGAGAGGAATCCTCCTGGGTTTCCAGATCCTGTACCAGAGAGTGACTCTGAGGTTCCGCCCT
H02-07 (H...    GCCCTCCCTCTGGTCTTGAGGGAGAAGAATCCTCCTGGGTTT------------CCAGAGAGTGACTCTGAGGGTCCGCCCT
H02-14 (H...    GCCCTCCCTCTGGTCTTGAGGGAGAAGAATCCTCCTGGGTTT------------CCAGAGAGTGACTCTGAGGGTCCGCCCT
H02-18 (H...    GCCCTCCCTCTGGTCTTGAGGGAGAAGAATCCTCCTGGGTTT------------CCAGAGAGTGACTCTGAGGGTCCGCCCT

..................................................................................................
```

```
             1477                                                                             1558
Untitled ... GCTCTCTGACACAATTAAGGGATAAAATCTCTGAAGAAATGAAGGGAAGACAATCCCTCGAATACTGATGAGTGGTTCCCTT
A11-01010... GCTCTCTGACACAATTAAGGGATAAAATCTCTGAAGGAGTGACGGGAAGACGATCCCTCGAATACTGATGAGTGGTTCCCTT
A11-126 (... GCTCTCTGACACAATTAAGGGATAAAATCTCTGAAGGAGTGACGGGAAGACGATCCCTCGAATACTGATGAGTGGTTCCCTT
A11-335 (... GCTCTCTGACACAATTAAGGGATAAAATCTCTGAAGGAGTGACGGGAAGACGATCCCTCGAATACTGATGAGTGGTTCCCTT
H02-07 (H... GCTCTCTGACACAATTAAGGGATGAAATCTGTGAGGAAATGAAGGGAAGACAATCCCTGGAATACTGATGAGTGGTTCCCTT
H02-14 (H... GCTCTCTGACACAATTAAGGGATGAAATCTGTGAGGAAATGAAGGGAAGACAATCCCTGGAATACTGATGAGTGGTTCCCTT
H02-18 (H... GCTCTCTGACACAATTAAGGGATGAAATCTGTGAGGAAATGAAGGGAAGACAATCCCTGGAATACTGATGAGTGGTTCCCTT

...............................................................................................

             1559                                                                             1640
Untitled ... TGACACCGGCAGCAGCCTTGGGCCCCGTGACTTTTCCTCTCAGGCCTTGTTCTCTGCTTCACACTCAATGTGCGTGGGGGTC
A11-01010... TGACACCGGCAGCAGCCTTGGG-CCCGTGACTTTTCCTCTCAGGCCTTGTTCTCTGCTTCACACTCAATGTGTGTGGGGGTC
A11-126 (... TGACACCGGCAGCAGCCTTGGG-CCCGTGACTTTTCCTCTCAGGCCTTGTTCTCTGCTTCACACTCAATGTGTGTGGGGGTC
A11-335 (... TGACACCGGCAGCAGCCTTGGG-CCCGTGACTTTTCCTCTCAGGCCTTGTTCTCTGCTTCACACTCAATGTGTGTGGGGGTC
H02-07 (H... TGACACTGGCAGCAGCCTTGGGCCCCGTGACTTTTCCTCTCAGGCCTTGTTCTCTGCTTCACACTCAATGTGCGTGGGGGTC
H02-14 (H... TGACACTGGCAGCAGCCTTGGGCCCCGTGACTTTTCCTCTCAGGCCTTGTTCTCTGCTTCACACTCAATGTGCGTGGGGGTC
H02-18 (H... TGACACTGGCAGCAGCCTTGGGCCCCGTGACTTTTCCTCTCAGGCCTTGTTCTCTGCTTCACACTCAATGTGCGTGGGGGTC

...............................................................................................

             1641                                                                             1722
Untitled ... TGAGTCCAGCACTTCTGAGTCCCTCAGCCTCCACTCAGGTCAGGACCAGAAGTCGCTGTTCCCTCCTCAGGGACTAGAATTT
A11-01010... TGAGTCCAGCACTTCTGAGTCTCTCAGCCTCCACTCAGGTCAGGACCAGAAGTCGCTGTTCCCTTCTCAG------------
A11-126 (... TGAGTCCAGCACTTCTGAGTCTCTCAGCCTCCACTCAGGTCAGGACCAGAAGTCGCTGTTCCCTTCTCAG------------
A11-335 (... TGAGTCCAGCACTTCTGAGTCTCTCAGCCTCCACTCAGGTCAGGACCAGAAGTCGCTGTTCCCTTCTCAG------------
H02-07 (H... TGAGTCCAGCTCTTCTGAGTCCCTCAGCCTCCACTCAGGTCAGGACCAGAAGTCGCTGTTCCCTCTTCAGGGACTAGAATTT
H02-14 (H... TGAGTCCAGCTCTTCTGAGTCCCTCAGCCTCCACTCAGGTCAGGACCAGAAGTCGCTGTTCCCTCTTCAGGGACTAGAATTT
H02-18 (H... TGAGTCCAGCTCTTCTGAGTCCCTCAGCCTCCACTCAGGTCAGGACCAGAAGTCGCTGTTCCCTCTTCAGGGACTAGAATTT

...............................................................................................

             1723                                                                             1804
Untitled ... TCCACGGAATAGAAGATTATCCCAGGTGCCTCTGTCCAGGCTGTTGTCTGGGTTCTGTGCTCCCTTCCCCACCCCAGGCATC
A11-01010... -----GGAATAGAAGATTATCCCAGGTGCCTGTGTCCAGGCTGGTGTCTGGGTTCTGTGCTCTCTTCCCCATCCCGGGTGTC
A11-126 (... -----GGAATAGAAGATTATCCCAGGTGCCTGTGTCCAGGCTGGTGTCTGGGTTCTGTGCTCTCTTCCCCATCCCGGGTGTC
A11-335 (... -----GGAATAGAAGATTATCCCAGGTGCCTGTGTCCAGGCTGGTGTCTGGGTTCTGTGCTCTCTTCCCCATCCCGGGTGTC
H02-07 (H... TCCACGGAATAGGAGATTATCCCAGGTGCCTCTGTCCAGGCTGTTGTCTGGGTTCTGTGCTCCCTTCCCCACCCCAGGCATC
H02-14 (H... TCCACGGAATAGGAGATTATCCCAGGTGCCTCTGTCCAGGCTGTTGTCTGGGTTCTGTGCTCCCTTCCCCACCCCAGGCATC
H02-18 (H... TCCACGGAATAGGAGATTATCCCAGGTGCCTCTGTCCAGGCTGTTGTCTGGGTTCTGTGCTCCCTTCCCCACCCCAGGCATC

...............................................................................................

             1805                                                                             1886
Untitled ... CTGTCAATTCTCAAGATGGCCACATGCGTGCTGGTGGAGTGTCCCATGACAGATGCAAAATGCCTGAATTTTCTGACTCTTC
A11-01010... CTGTCCATTCTCAAGATGGCCACATGCGTGCTGGTGGAGTGTCCCATGACAGATGCAAAATGCCTGAATTTTCTGACTCTTC
A11-126 (... CTGTCCATTCTCAAGATGGCCACATGCGTGCTGGTGGAGTGTCCCATGACAGATGCAAAATGCCTGAATTTTCTGACTCTTC
A11-335 (... CTGTCCATTCTCAAGATGGCCACATGCGTGCTGGTGGAGTGTCCCATGACAGATGCAAAATGCCTGAATTTTCTGACTCTTC
H02-07 (H... CTGTCAATTCTCAAGATGGCCACATGCGTGCTGGTGGAGTGTCCCATGACAGATGCAAAATGCCTGAATTTTCTGACTCTTC
H02-14 (H... CTGTCAATTCTCAAGATGGCCACATGCGTGCTGGTGGAGTGTCCCATGACAGATGCAAAATGCCTGAATTTTCTGACTCTTC
H02-18 (H... CTGTCAATTCTCAAGATGGCCACATGCGTGCTGGTGGAGTGTCCCATGACAGATGCAAAATGCCTGAATTTTCTGACTCTTC

...............................................................................................

             1887                                                                             1968
Untitled ... CCGTCAGACCCCCCCCAAGACACATATGACCCACCACCCCATCTCTGACCATGAGGCCACCCTGAGGTGCTGGGCCCTGGGC
A11-01010... CCGTCAGA-CCCCCCCAAGACACATATGACCCACCACCCCATCTCTGACCATGAGGCCACCCTGAGGTGCTGGGCCCTGGGC
A11-126 (... CCGTCAGA-CCCCCCCAAGACACATATGACCCACCACCCCATCTCTGACCATGAGGCCACCCTGAGGTGCTGGGCCCTGGGC
A11-335 (... CCGTCAGA-CCCCCCCAAGACACATATGACCCACCACCCCATCTCTGACCATGAGGCCACCCTGAGGTGCTGGGCCCTGGGC
H02-07 (H... CCGTCAGA-CCCCCCCAAGACACATATGACCCACCACCCCATCTCTGACCATGAGGCCACCCTGAGGTGCTGGGCCCTGGGC
H02-14 (H... CCGTCAGA-CCCCCCCAAGACACATATGACCCACCACCCCATCTCTGACCATGAGGCCACCCTGAGGTGCTGGGCCCTGGGC
H02-18 (H... CCGTCAGACCCCCCCCAAGACACATATGACCCACCACCCCATCTCTGACCATGAGGCCACCCTGAGGTGCTGGGCCCTGGGC

...............................................................................................
```

```
                1969                                                                            2050
Untitled ...    TTCTACCCTGCGGAGATCACACTGACCTGGCAGCGGGATGGGGAGGACCAGACCCAGGACACGGAGCTCGTGGAGACCAGGC
A11-01010...    TTCTACCCTGCGGAGATCACACTGACCTGGCAGCGGGATGGGGAGGACCAGACCCAGGACACGGAGCTCGTGGAGACCAGGC
A11-126 (...    TTCTACCCTGCGGAGATCACACTGACCTGGCAGCGGGATGGGGAGGACCAGACCCAGGACACGGAGCTCGTGGAGACCAGGC
A11-335 (...    TTCTACCCTGCGGAGATCACACTGACCTGGCAGCGGGATGGGGAGGACCAGACCCAGGACACGGAGCTCGTGGAGACCAGGC
H02-07 (H...    TTCTACCCTGCGGAGATCACACTGACCTGGCAGCGGGATGGGGAGGACCAGACCCAGGACACGGAGCTCGTGGAGACCAGGC
H02-14 (H...    TTCTACCCTGCGGAGATCACACTGACCTGGCAGCGGGATGGGGAGGACCAGACCCAGGACACGGAGCTCGTGGAGACCAGGC
H02-18 (H...    TTCTACCCTGCGGAGATCACACTGACCTGGCAGCGGGATGGGGAGGACCAGACCCAGGACACGGAGCTCGTGGAGACCAGGC

..................................................................................................

                2051                                                                            2132
Untitled ...    CTGCAGGGGATGGAACCTTCCAGAAGTGGGCGGCTGTGGTGGTGCCTTCTGGAGAGGAGCAGAGATACACCTGCCATGTGCA
A11-01010...    CTGCAGGGGATGGAACCTTCCAGAAGTGGGCGGCTGTGGTGGTGCCTTCTGGAGAGGAGCAGAGATACACCTGCCATGTGCA
A11-126 (...    CTGCAGGGGATGGAACCTTCCAGAAGTGGGCGGCTGTGGTGGTGCCTTCTGGAGAGGAGCAGAGATACACCTGCCATGTGCA
A11-335 (...    CTGCAGGGGATGGAACCTTCCAGAAGTGGGCGGCTGTGGTGGTGCCTTCTGGAGAGGAGCAGAGATACACCTGCCATGTGCA
H02-07 (H...    CTGCAGGGGATGGAACCTTCCAGAAGTGGGCGGCTGTGGTGGTGCCTTCTGGAGAGGAGCAGAGATACACCTGCCATGTGCA
H02-14 (H...    CTGCAGGGGATGGAACCTTCCAGAAGTGGGCGGCTGTGGTGGTGCCTTCTGGAGAGGAGCAGAGATACACCTGCCATGTGCA
H02-18 (H...    CTGCAGGGGATGGAACCTTCCAGAAGTGGGCGGCTGTGGTGGTGCCTTCTGGAGAGGAGCAGAGATACACCTGCCATGTGCA

..................................................................................................

                2133                                                                            2214
Untitled ...    GCATGAGGGTCTGCCCGAGCCCCTCACCCTGAGATGGGGTAAGGAGGGAGATGGGGGTGTCATGTCTCTTAGGGAAAGCCGG
A11-01010...    GCATGAGGGTCTGCCCAAGCCCCTCACCCTGAGATGGGGTAAGGAGGGAGATGGGGGTGTCATGTCTCTTAGGGAAAGCAGG
A11-126 (...    GCATGAGGGTCTGCCCGAGCCCCTCACCCTGAGATGGGGTAAGGAGGGAGATGGGGGTGTCATGTCTCTTAGGGAAAGCAGG
A11-335 (...    GCATGAGGGTCTGCCCGAGCCCCTCACCCTGAGATGGGGTAAGGAGGGAGATGGGGGTGTCATGTCTCTTAGGGAAAGCCGG
H02-07 (H...    GCATGAGGGTCTGCCCGAGCCCCTCACCCTGAGATGGGGTAAGGAGGGAGATGGGGGTGTCATGTCTCTTAGGGAAAGCCGG
H02-14 (H...    GCATGAGGGTCTGCCCGAGCCCCTCACCCTGAGATGGGGTAAGGAGGGAGATGGGGGTGTCATGTCTCTTAGGGAAAGCCGG
H02-18 (H...    GCATGAGGGTCTGCCCGAGCCCCTCACCCTGAGATGGGGTAAGGAGGGAGATGGGGGTGTCATGTCTCTTAGGGAAAGCCGG

..................................................................................................

                2215                                                                            2296
Untitled ...    AGCCTCTCTGGAGAGCTTTAGCAGGGTCAGGGTCCCTCACCTTCCCCCCTTTTCCCAGAGCCATCTTCCCAGCCCACCGTCC
A11-01010...    AGCCTCTCTGGAGACCTTTAGCAGGGTCAGGGCCCCTCACCTTCCCCTCTTTTCCCAGAGCTGTCTTCCCAGCCCACCATCC
A11-126 (...    AGCCTCTCTGGAGACCTTTAGCAGGGTCAGGGCCCCTCACCTTCCCCTCTTTTCCCAGAGCTGTCTTCCCAGCCCACCATCC
A11-335 (...    AGCCTCTCTGGAGAGCTTTAGCAGGGTCAGGGTCCCTCACCTTCCCCCCTTTTCCCAGAGCCATCTTCCCAGCCCACCGTCC
H02-07 (H...    AGCCTCTCTGGAGAGCTTTAGCAGGGTCAGGGTCCCTCACCTTCCCCCCTTTTCCCAGAGCCATCTTCCCAGCCCACCGTCC
H02-14 (H...    AGCCTCTCTGGAGAGCTTTAGCAGGGTCAGGGTCCCTCACCTTCCCCCCTTTTCCCAGAGCCATCTTCCCAGCCCACCGTCC
H02-18 (H...    AGCCTCTCTGGAGAGCTTTAGCAGGGTCAGGGTCCCTCACCTTCCCCCCTTTTCCCAGAGCCATCTTCCCAGCCCACCGTCC

..................................................................................................

                2297                                                                            2378
Untitled ...    CCATCGTGGGCATCGTTGCTGGCCTGGTTCTACTTGTAGCTGTGGTCACTGGAGCTGTGGTCGCTGCTGTAATGTGGAGGAA
A11-01010...    CCATCGTGGGCATCATTGCTGGCCTGGTTCTCCTTGGAGCTGTGATCACTGGAGCTGTGGTCGCTGCCGTGATGTGGAGGAG
A11-126 (...    CCATCGTGGGCATCATTGCTGGCCTGGTTCTCCTTGGAGCTGTGATCACTGGAGCTGTGGTCGCTGCCGTGATGTGGAGGAG
A11-335 (...    CCATCGTGGGCATCGTTGCTGGCCTGGTTCTACTTGTAGCTGTGGTCACTGGAGCTGTGGTCGCTGCTGTAATGTGGAGGAA
H02-07 (H...    CCATCGTGGGCATCGTTGCTGGCCTGGTTCTACTTGTAGCTGTGGTCACTGGAGCTGTGGTCGCTGCTGTAATGTGGAGGAA
H02-14 (H...    CCATCGTGGGCATCGTTGCTGGCCTGGTTCTACTTGTAGCTGTGGTCACTGGAGCTGTGGTCGCTGCTGTAATGTGGAGGAA
H02-18 (H...    CCATCGTGGGCATCGTTGCTGGCCTGGTTCTACTTGTAGCTGTGGTCACTGGAGCTGTGGTCGCTGCTGTAATGTGGAGGAA

..................................................................................................

                2379                                                                            2460
Untitled ...    GAAGAGCTCAGGTAAGGAAGGGGTGAGGAGTGTGGTCTGAGATTTCTTGTCTCACTGAGAGTTCCAAGCCCCAGGTAGAAAT
A11-01010...    GAAGAGCTCAGGTGGAGAAGGGGTGAAAGGTGGGGTCTGAGATTTCTTGTCTCACTGAGGGTTCCAAGCCCCAGCTAGAAAT
A11-126 (...    GAAGAGCTCAGGTGGAGAAGGGGTGAAAGGTGGGGTCTGAGATTTCTTGTCTCACTGAGGGTTCCAAGCCCCAGCTAGAAAT
A11-335 (...    GAAGAGCTCAGGTAAGGAAGGGGTGAGGAGTGTGGTCTGAGATTTCTTGTCTCACTGAGAGTTCCAAGCCCCAGGTAGAA--
H02-07 (H...    GAAGAGCTCAGGTAAGGAAGGGGTGAGGAGTGTGGTCTGAGATTTCTTGTCTCACTGAGAGTTCCAAGCCCCAGGTAGAA--
H02-14 (H...    GAAGAGCTCAGGTAAGGAAGGGGTGAGGAGTGTGGTCTGAGATTTCTTGTCTCACTGAGAGTTCCAAGCCCCAGGTAGAA--
H02-18 (H...    GAAGAGCTCAGGTAAGGAAGGGGTGAGGAGTGTGGTCTGAGATTTCTTGTCTCACTGAGAGTTCCAAGCCCCAGGTAGAA--

..................................................................................................
```

```
             2461                                                                          2542
Untitled ... GTGCCCTGCCTGGTTACTGGGAAGCACCATCCACACTCATGGGCCTACCCAGCCTGGGCCCTGTGTGCCAGCACTTACTCTT
A11-01010... GTGCCCTGTCTCATTACTGGGAAGCACCTTCCACAATCATGGGCCGACCCAGCCTGGGCCCTGTGTGCCAGCACTTACTCTT
A11-126 (... GTGCCCTGTCTCATTACTGGGAAGCACCTTCCACAATCATGGGCCGACCCAGCCTGGGCCCTGTGTGCCAGCACTTACTCTT
A11-335 (... GTGCCCTGCCTGGTTACTGGGAAGCACCATCCACACTCATGGGCCTACCCAGCCTGGGCCCTGTGTGCCAGCACTTACTCTT
H02-07 (H... GTGCCCTGCCTGGTTACTGGGAAGCACCATCCACACTCATGGGCCTACCCAGCCTGGGCCCTGTGTGCCAGCACTTACTCTT
H02-14 (H... GTGCCCTGCCTGGTTACTGGGAAGCACCATCCACACTCATGGGCCTACCCAGCCTGGGCCCTGTGTGCCAGCACTTACTCTT
H02-18 (H... GTGCCCTGCCTGGTTACTGGGAAGCACCATCCACACTCATGGGCCTACCCAGCCTGGGCCCTGTGTGCCAGCACTTACTCTT

...............................................................................................

             2543                                                                          2624
Untitled ... TTGTAAAGCACCTGTTACAATGAGGGACAGATTTATCACCTTGATGACTGTGGTGATGGGACCTGATCCCAGCAGTCACAAG
A11-01010... TTGTAAAGCACCTGTTAAAATGAAGGACAGATTTATCACCTTGATTACGGCGGTGATGGGACCTGATACCAGCAGTCACAAG
A11-126 (... TTGTAAAGCACCTGTTAAAATGAAGGACAGATTTATCACCTTGATTACGGCGGTGATGGGACCTGATACCAGCAGTCACAAG
A11-335 (... TTGTAAAGCACCTGTTACAATGAGGGACAGATTTATCACCTTGATGACTGTGGTGATGGGACCTGATCCCAGCAGTCACAAG
H02-07 (H... TTGTAAAGCACCTGTTACAATGAGGGACAGATTTATCACCTTGATGACTGTGGTGATGGGACCTGATCCCAGCAGTCACAAG
H02-14 (H... TTGTAAAGCACCTGTTACAATGAGGGACAGATTTATCACCTTGATGACTGTGGTGATGGGACCTGATCCCAGCAGTCACAAG
H02-18 (H... TTGTAAAGCACCTGTTACAATGAGGGACAGATTTATCACCTTGATGACTGTGGTGATGGGACCTGATCCCAGCAGTCACAAG

...............................................................................................

             2625                                                                          2706
Untitled ... TCACAGGGGAAGGTCCCCGAGGACAGACCTCAGAAGGGCGGTTGGTCCAGGACCCACATCTGCTTTCTTCATGTTTCCTGAT
A11-01010... TCACAGGGGAAGGTCCCTGAGGACAGACCTCAGGAGGGCTATTGGTCCAGGACCCACACCTGCTTTCTTCATGTTTCCTGAT
A11-126 (... TCACAGGGGAAGGTCCCTGAGGACAGACCTCAGGAGGGCTATTGGTCCAGGACCCACACCTGCTTTCTTCATGTTTCCTGAT
A11-335 (... TCACAGGGGAAGGTCCCCGAGGACAGACCTCAGAAGGGCGGTTGGTCCAGGACCCACATCTGCTTTCTTCATGTTTCCTGAT
H02-07 (H... TCACAGGGGAAGGTCCCCGAGGACAGACCTCAGAAGGGCGGTTGGTCCAGGACCCACATCTGCTTTCTTCATGTTTCCTGAT
H02-14 (H... TCACAGGGGAAGGTCCCCGAGGACAGACCTCAGAAGGGCGGTTGGTCCAGGACCCACATCTGCTTTCTTCATGTTTCCTGAT
H02-18 (H... TCACAGGGGAAGGTCCCCGAGGACAGACCTCAGAAGGGCGGTTGGTCCAGGACCCACATCTGCTTTCTTCATGTTTCCTGAT

...............................................................................................

             2707                                                                          2788
Untitled ... CCCGCCCTGGGTCTGCAGTTGCACATTTCTGGAAACTTCTCTGGGGTCCGAGACTTGGAGGTTCCTCTAGGACCTTAAGGCC
A11-01010... CCCGCCCTGGGTCTGCAGTCACACATTTCTGGAAACTTCTCTGGGGTCCAAGACTAGGAGGTTCCTCTAGGACCTTAAGGCC
A11-126 (... CCCGCCCTGGGTCTGCAGTCACACATTTCTGGAAACTTCTCTGGGGTCCAAGACTAGGAGGTTCCTCTAGGACCTTAAGGCC
A11-335 (... CCCGCCCTGGGTCTGCAGTTGCACATTTCTGGAAACTTCTCTGGGGTCCGAGACTTGGAGGTTCCTCTAGGACCTTAAGGCC
H02-07 (H... CCCGCCCTGGGTCTGCAGTTGCACATTTCTGGAAACTTCTCTGGGGTCCGAGACTTGGAGGTTCCTCTAGGACCTTATGGCC
H02-14 (H... CCCGCCCTGGGTCTGCAGTTGCACATTTCTGGAAACTTCTCTGGGGTCCGAGACTTGGAGGTTCCTCTAGGACCTTATGGCC
H02-18 (H... CCCGCCCTGGGTCTGCAGTTGCACATTTCTGGAAACTTCTCTGGGGTCCGAGACTTGGAGGTTCCTCTAGGACCTTATGGCC

...............................................................................................

             2789                                                                          2870
Untitled ... CTGGCTCCTTTCTGGCATCTCACAGGACATTTTCTTCCCACAGATAGAAAAGGAGGGAGCTACACTCAGGCTGCAAGTAAGT
A11-01010... CTGGCTCCTTTCTGGTATCTCACAGGACATTTTCTTCCCACAGATAGAAAAGGAGGGAGTTACACTCAGGCTGCAAGTAAGT
A11-126 (... CTGGCTCCTTTCTGGTATCTCACAGGACATTTTCTTCCCACAGATAGAAAAGGAGGGAGTTACACTCAGGCTGCAAGTAAGT
A11-335 (... CTGGCTCCTTTCTGGTATCTCACAGGACATTTTCTTCCCACAGATAGAAAAGGAGGGAGTTACACTCAGGCTGCAAGTAAGT
H02-07 (H... CTGGCTTCTTTCTGGCATCTCACAGGACATTTTCTTCCCACAGATAGAAAAGGAGGGAGCTACTCTCAGGCTGCAAGTAAGT
H02-14 (H... CTGGCTTCTTTCTGGCATCTCACAGGACATTTTCTTCCCACAGATAGAAAAGGAGGGAGCTACTCTCAGGCTGCAAGTAAGT
H02-18 (H... CTGGCTTCTTTCTGGCATCTCACAGGACATTTTCTTCCCACAGATAGAAAAGGAGGGAGCTACTCTCAGGCTGCAAGTAAGT

...............................................................................................

             2871                                                                          2952
Untitled ... ATGAAGGAGGCTGATCCCTGAAATCCTTTGGATATTGTGTTTGGGAGCCCATGGGGGAGCTCACCCACCCCACAATTCCTCC
A11-01010... ATGAAGGAGGCTGATGCCTGAGGTCCTTGGGATATTGTGTTTGGGAGCCCATGGGGGAGCTCACCCACCCCACAATTCCTCC
A11-126 (... ATGAAGGAGGCTGATGCCTGAGGTCCTTGGGATATTGTGTTTGGGAGCCCATGGGGGAGCTCACCCACCCCACAATTCCTCC
A11-335 (... ATGAAGGAGGCTGATGCCTGAGGTCCTTGGGATATTGTGTTTGGGAGCCCATGGGGGAGCTCACCCACCCCACAATTCCTCC
H02-07 (H... ATGAAGGAGGCTGATCCCTGAAATCCTTTGGATATTGTGTTTGGGAGCCCATGGGGGAGCTCACCCACCCCACAATTCTTCC
H02-14 (H... ATGAAGGAGGCTGATCCCTGAAATCCTTTGGATATTGTGTTTGGGAGCCCATGGGGGAGCTCACCCACCCCACAATTCTTCC
H02-18 (H... ATGAAGGAGGCTGATCCCTGAAATCCTTTGGATATTGTGTTTGGGAGCCCATGGGGGAGCTCACCCACCCCACAATTCTTCC

...............................................................................................
```

```
               2953                                                                            3034
Untitled ...   TCTAGCCACATCTACTGTGGGATCTGACCAGGTCCTGTTTTTATTCTACCCCAGGCAGCAACAGTGCCCAGGGCTCTGATGT
A11-01010...   TCTAGCCACATCTTCTGTGGGATCTGACCAGGTTCTGTTTTTGTTCTACCCCAGGCAGTGACAGTGCCCAGGGCTCTGATGT
A11-126 (...   TCTAGCCACATCTTCTGTGGGATCTGACCAGGTTCTGTTTTTGTTCTACCCCAGGCAGTGACAGTGCCCAGGGCTCTGATGT
A11-335 (...   TCTAGCCACATCTTCTGTGGGATCTGACCAGGTTCTGTTTTTGTTCTACCCCAGGCAGTGACAGTGCCCAGGGCTCTGATGT
H02-07 (H...   TCTAGCCACATCTACTGTGGGATCTGACCAGGTCCTGTTTTTATTCTACTCCAGGCAGCAACAGTGCCCAGGGCTCTGATGT
H02-14 (H...   TCTAGCCACATCTACTGTGGGATCTGACCAGGTCCTGTTTTTATTCTACTCCAGGCAGCAACAGTGCCCAGGGCTCTGATGT
H02-18 (H...   TCTAGCCACATCTACTGTGGGATCTGACCAGGTCCTGTTTTTATTCTACTCCAGGCAGCAACAGTGCCCAGGGCTCTGATGT

..................................................................................................

               3035                                                                            3116
Untitled ...   GTCTCTCACAGCTTGTAAAGGTGAGACCTTGGAGGGCCTGATGTGTGTTGGATGTTGGGGCGGAACAGTGGACACAGCTGTG
A11-01010...   GTCTCTCACAGCTTGTAAAGGTGAGAGCTTGGAGGGCCTGATGTGTGTTGGGTGTT-GGGCGGAACAGTGGACACAGCTGTG
A11-126 (...   GTCTCTCACAGCTTGTAAAGGTGAGAGCTTGGAGGGCCTGATGTGTGTTGGGTGTT-GGGCGGAACAGTGGACACAGCTGTG
A11-335 (...   GTCTCTCACAGCTTGTAAAGGTGAGAGCTTGGAGGGCCTGATGTGTGTTGGGTGTT-GGGCGGAACAGTGGACACAGCTGTG
H02-07 (H...   GTCTCTCACGGCTTG-AAAGGTGAGACCTTGGGGGGCCTGATGTGTGGGGGATGTTGGGGGGGAACAGTGGACACAGCTGTG
H02-14 (H...   GTCTCTCACGGCTTG-AAAGGTGAGACCTTGGGGGGCCTGATGTGTGGGGGATGTTGGGGGGGAACAGTGGACACAGCTGTG
H02-18 (H...   GTCTCTCACGGCTTG-AAAGGTGAGACCTTGGGGGGCCTGATGTGTGGGGGATGTTGGGGGGGAACAGTGGACACAGCTGTG

..................................................................................................

               3117                                                                            3198
Untitled ...   CTATGGGGTTTCTTTGAATTTGATGTATTGAGCATGCGATGGGCTGTCCAAAGTGTCACCCATCACTGTGACAGATATGAAT
A11-01010...   CTATGGGGTTTCTTTGCATTGGATGTATTGAGCATGCGATGGGCTGTTTAAGGTGTGACCCCTCACTGTGATGGATATGAAT
A11-126 (...   CTATGGGGTTTCTTTGCATTGGATGTATTGAGCATGCGATGGGCTGTTTAAGGTGTGACCCCTCACTGTGATGGATATGAAT
A11-335 (...   CTATGGGGTTTCTTTGCATTGGATGTATTGAGCATGCGATGGGCTGTTTAAGGTGTGACCCCTCACTGTGATGGATATGAAT
H02-07 (H...   CTATGGGG-TTCTTTGAATTTGATGTTTTGAGCATGCGATGGGCTG-CCAAAGTGTCATCCATTACTGGGACAGATATGAAT
H02-14 (H...   CTATGGGG-TTCTTTGAATTTGATGTTTTGAGCATGCGATGGGCTG-CCAAAGTGTCATCCATTACTGGGACAGATATGAAT
H02-18 (H...   CTATGGGG-TTCTTTGAATTTGATGTTTTGAGCATGCGATGGGCTG-CCAAAGTGTCATCCATTACTGGGACAGATATGAAT

..................................................................................................

               3199                                                                            3280
Untitled ...   TTGTTCATGAATATTTTTTTCTATAGTGTGAGACAGCTGCCTTGTGTGGGACTGAGAGGCAAGAGTTGTTCCTGCCCTTCCC
A11-01010...   TTGTTCATGAATATTTTTTTCTATAGTGTGAGACAGCTGCCTTGTGTGGGACTGAGAGGCAAGAGTTGTTCCTGCCCTTCCC
A11-126 (...   TTGTTCATGAATATTTTTTTCTATAGTGTGAGACAGCTGCCTTGTGTGGGACTGAGAGGCAAGAGTTGTTCCTGCCCTTCCC
A11-335 (...   TTGTTCATGAATATTTTTTTCTATAGTGTGAGACAGCTGCCTTGTGTGGGACTGAGAGGCAAGAGTTGTTCCTGCCCTTCCC
H02-07 (H...   TTGTTCATGAATA-TTTTTTCTATAGTGTGAGACAGCTGCCTTGTGTGGGACTGAGAGGCAAGAGTTGTTCCTG-CCTTCCC
H02-14 (H...   TTGTTCATGAATA-TTTTTTCTATAGTGTGAGACAGCTGCCTTGTGTGGGACTGAGAGGCAAGAGTTGTTCCTG-CCTTCCC
H02-18 (H...   TTGTTCATGAATA-TTTTTTCTATAGTGTGAGACAGCTGCCT----------------------------------------

..................................................................................................

               3281                                                                            3362
Untitled ...   TTTGTGACTTGAAGAACCCTGACTTTCTTTCTACAAAGGCACCTGAATGTGTCTGTGTTCCTGTAGGCATAATGTGAGGAGG
A11-01010...   TTTGTGACTTGAAGAACCCTGACTTTGTTTCTGCAAAGGCACCTGCATGTGTCTGTGTTCGTGTAGGCATAATGTGAGGAGG
A11-126 (...   TTTGTGACTTGAAGAACCCTGACTTTGTTTCTGCAAAGGCACCTGCATGTGTCTGTGTTCGTGTAGGCATAATGTGAGGAGG
A11-335 (...   TTTGTGACTTGA----------------------------------------------------------------------
H02-07 (H...   TTTGTGACTTGAAGAACCCTGACTTTCTTTCTACAAAGGCACCTGAATGTGTCTGTGTTCCTGTAGGCATAATGTGTGGAGG
H02-14 (H...   TTTGTGACTTGAAGAACCCTGACTTTCTTTCTACAAAGGCACCTGAATGTGTCTGTGTTCCTGTAGGCATAATGTGTGGAGG
H02-18 (H...   ----------------------------------------------------------------------------------

..................................................................................................

               3363                                                                            3444
Untitled ...   AGGGGAGACCAACCCACCCCCATGTCCACCATGACCCTCTTCCCCACGCTGATCTGTGTTCCCTCCCCAATCATCTTTCCTG
A11-01010...   TGGGGAGACCACCCCACCCCGATGTCCACCATGACCCTCTT-CCCACGCTGACCTGTGCTCCCTCTCCAATCATCTTTCCTG
A11-126 (...   TGGGGAGACCACCCCACCCCGATGTCCACCATGACCCT--------------------------------------------
A11-335 (...   ----------------------------------------------------------------------------------
H02-07 (H...   AGGGGAGACCAACCCACCCCTCATGTCCACCATGACCCTCTTCCCCACGCTGATCTGTGTTCCCTCCCCAATCATCTTTCCTG
H02-14 (H...   AGGGGAGACCAACCCACCCCTCATGTCCACCATGACCCTCTTCCCCACGCTGATCTGTGTTCCCTCCCCAATCATCTTTCCTG
H02-18 (H...   ----------------------------------------------------------------------------------

..................................................................................................
```

```
               3445                                                                          3526
Untitled ...  TTCCAGAGAGGAGGGGCTGAGATGTCTCCATCTTTTTCTCAACTTTATGGTGCACTGAGCTGTAACTTCTTACTTCCCTCTT
A11-01010...  TTCCAGAGAGGTGGGGCTGAGGTGTCTCCATCTCTGTCTCAACTTCATGGTGCACTGAGCTGTAACTTCTTCCTTCCCTATT
A11-126 (...  ----------------------------------------------------------------------------------
A11-335 (...  ----------------------------------------------------------------------------------
H02-07 (H...  TTCCAGAGAGGAGGGGCTGAGATGTCTCCATCTTTTTCTCAACTTTAT-GTGCACTGAGCTGTAA-TTCTTACTTCCCTCTT
H02-14 (H...  TTCCAGAGAGGAGGGGCTGAGATGTCTCCATCTTTTTCTCAACTTTAT-GTGCACTGAGCTGTAA-TTCTTACTTCCCTCTT
H02-18 (H...  ----------------------------------------------------------------------------------

.............................................................................................

               3527 3535
Untitled ...  AAAATTAGA
A11-01010...  AAAA-----
A11-126 (...  ---------
A11-335 (...  ---------
H02-07 (H...  AAAATTAGA
H02-14 (H...  AAAATTAGA
H02-18 (H...  ---------

.............................................................................................
```
